# *N*-[(3-Ethyl­phen­yl)carbamo­thio­yl]-2,2-di­phenyl­acetamide

**DOI:** 10.1107/S1600536813014268

**Published:** 2013-06-08

**Authors:** Mohd Sukeri Mohd Yusof, Nur Rafikah Razali, Suhana Arshad, Azhar Abdul Rahman, Ibrahim Abdul Razak

**Affiliations:** a Department of Chemical Sciences, Faculty of Science and Technology, Universiti Malaysia Terengganu, Mengabang Telipot, 21030 Kuala Terengganu, Malaysia; b School of Physics, Universiti Sains Malaysia, 11800 USM, Penang, Malaysia

## Abstract

In the title mol­ecule, C_23_H_22_N_2_OS, the di­phenyl­acetyl and ethyl­benzene groups adopt a *trans*–*cis* conformation, respectively, with respect to the S atom across the (S=)C—N bonds. This conformation is stabilized by an intra­molecular N—H⋯O hydrogen bond and a weak C—H⋯S hydrogen bond. The ethyl-substituted benzene ring forms dihedral angles of 87.53 (15) and 73.94 (15)° with the phenyl rings. In the crystal, N—H⋯O hydrogen bonds link mol­ecules into chains along [100]. A weak C—H⋯π inter­action is also observed.

## Related literature

For the biological activity of carbonyl­thio­urea derivatives, see: Zhong *et al.* (2008 ▶); Saeed *et al.* (2010 ▶). For related structures, see: Yusof *et al.* (2012*a* ▶,*b* ▶). For hydrogen-bond motifs, see: Bernstein *et al.* (1995 ▶). For the stability of the temperature controller used for the data collection, see: Cosier & Glazer (1986 ▶). For standard bond lenths, see: Allen *et al.* (1987 ▶).
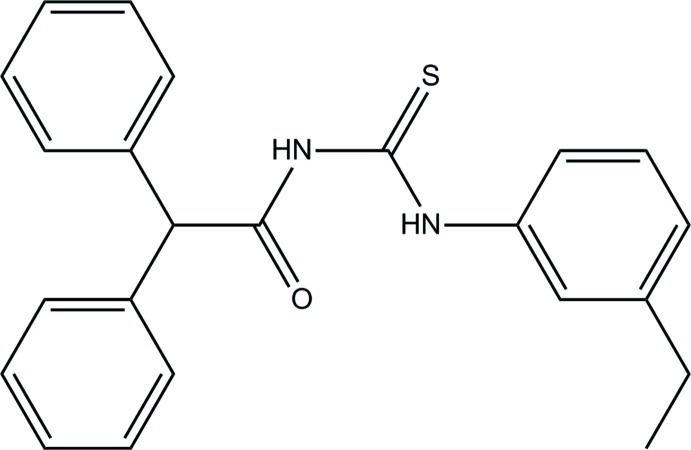


## Experimental

### 

#### Crystal data

C_23_H_22_N_2_OS
*M* *_r_* = 374.49
Orthorhombic, 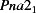

*a* = 10.0608 (2) Å
*b* = 17.9092 (5) Å
*c* = 10.8495 (3) Å
*V* = 1954.87 (9) Å^3^
*Z* = 4
Mo *K*α radiation
μ = 0.18 mm^−1^
*T* = 100 K
0.26 × 0.23 × 0.09 mm

#### Data collection

Bruker SMART APEXII CCD area-detector diffractometer
Absorption correction: multi-scan (*SADABS*; Bruker, 2009 ▶) *T*_min_ = 0.955, *T*_max_ = 0.983
11560 measured reflections
4121 independent reflections
2885 reflections with *I* > 2σ(*I*)
*R*_int_ = 0.071

#### Refinement

*R*[*F* ^2^ > 2σ(*F* ^2^)] = 0.057
*wR*(*F* ^2^) = 0.098
*S* = 0.99
4121 reflections
253 parameters
1 restraint
H atoms treated by a mixture of independent and constrained refinement
Δρ_max_ = 0.44 e Å^−3^
Δρ_min_ = −0.32 e Å^−3^
Absolute structure: Flack (1983 ▶), 1761 Friedel pairs
Flack parameter: 0.17 (9)

### 

Data collection: *APEX2* (Bruker, 2009 ▶); cell refinement: *SAINT* (Bruker, 2009 ▶); data reduction: *SAINT*; program(s) used to solve structure: *SHELXTL* (Sheldrick, 2008 ▶); program(s) used to refine structure: *SHELXTL*; molecular graphics: *SHELXTL*; software used to prepare material for publication: *SHELXTL* and *PLATON* (Spek, 2009 ▶).

## Supplementary Material

Crystal structure: contains datablock(s) global, I. DOI: 10.1107/S1600536813014268/lh5616sup1.cif

Structure factors: contains datablock(s) I. DOI: 10.1107/S1600536813014268/lh5616Isup2.hkl

Supplementary material file. DOI: 10.1107/S1600536813014268/lh5616Isup3.cml

Additional supplementary materials:  crystallographic information; 3D view; checkCIF report

## Figures and Tables

**Table 1:** Hydrogen-bond geometry (Å, °)

*D*—H⋯*A*	*D*—H	H⋯*A*	*D*⋯*A*	*D*—H⋯*A*
N2—H1*N*2⋯O1	0.90 (3)	1.82 (3)	2.630 (3)	148 (3)
C21—H21*A*⋯S1	0.95	2.61	3.255 (3)	126
N1—H1*N*1⋯O1^i^	0.82 (3)	2.25 (3)	3.023 (3)	157 (3)
C7—H7*A*⋯*Cg* ^ii^	1.00	2.87	3.844 (3)	166

Symmetry codes: (i) 
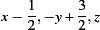
; (ii) 
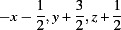
.

**Fig. 1.**
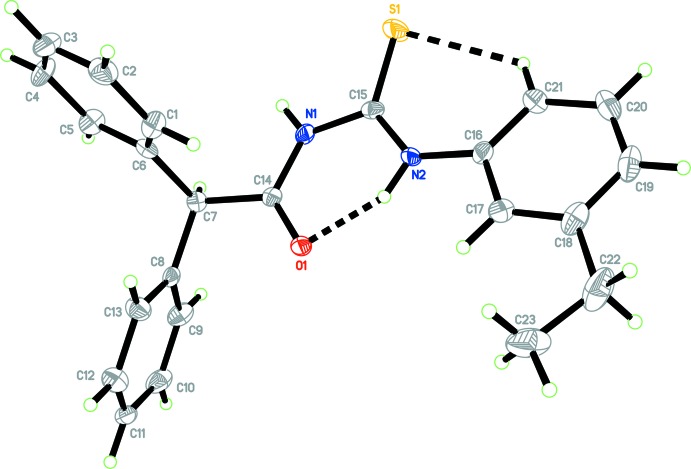
The molecular structure of the title compound shown with 50% probability displacement ellipsoids. Dashed lines represent intramolecular hydrogen bonds.

**Fig. 2.**
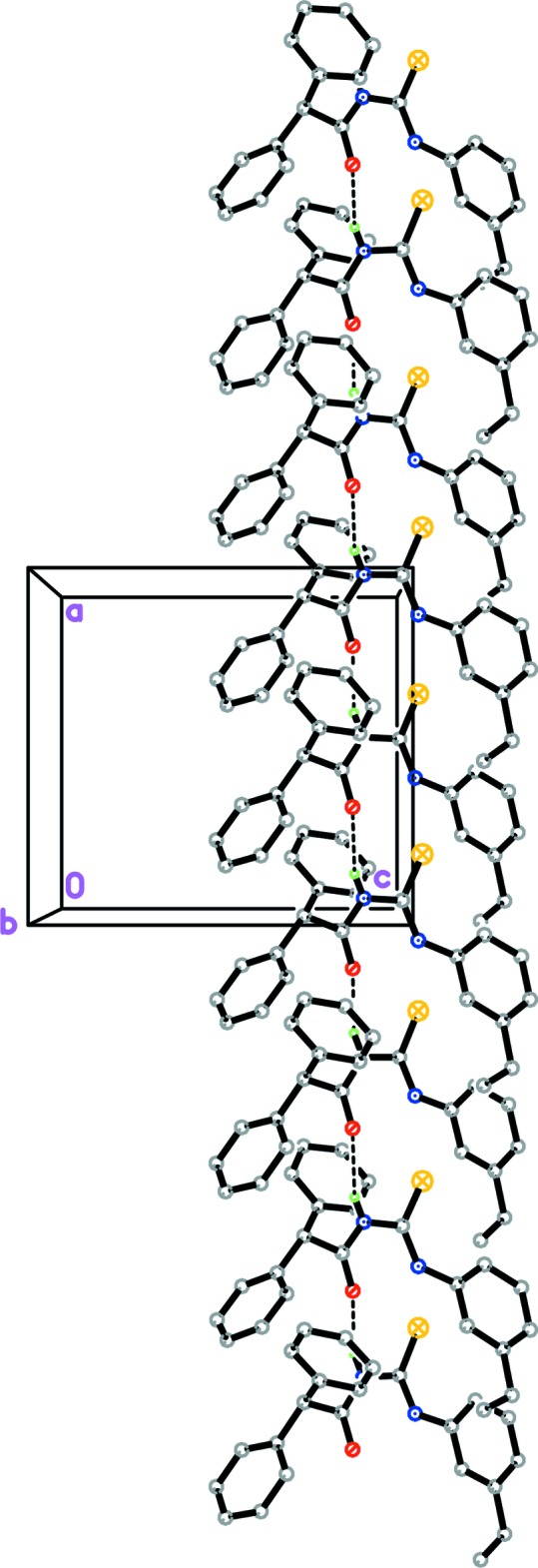
The crystal packing of the title compound. The H atoms not involved in the intermolecular hydrogen bonds (dashed lines) have been omitted for clarity.

## supplementary crystallographic information

### Comment

Carbonylthiourea derivatives have been explored because they are reported to posses biological activities such as antibacterial, anti-fungal and antiviral (Zhong *et al.*, 2008; Saeed *et al.*, 2010). The crystal structure of the title compound is presented herein.

The molecular structure is shown in Fig. 1. The diphenylacetyl and ethylbenzene groups adopt a *trans*-*cis* configuration, respectively, with respect to the sulfur atom across the (S═)C–N bonds. Intramolecular N2—H1N2···O1 and C21—H21A···S1 hydrogen bonds result in two *S*(6) graph-set motifs (Bernstein *et al.*, 1995). The ethyl-substituted benzene ring (C16–C21) forms dihedral angles of 87.53 (15) and 73.94 (15)°, respectively with the C1–C6 and C8–C13 rings. The bond lengths (Allen *et al.*, 1987) and angles are within normal ranges and comparable to related structures (Yusof *et al.*, 2012*a*,*b*).

In the crystal molecules are linked into one-dimensional chains along the [100] via intermolecular N1—H1N1···O16^i^ hydrogen bonds (Table 1). In addition, a weak C7—H7A···*Cg*^ii^ interaction is observed (*Cg* is the centroid of C8–C13).

### Experimental

An acetone (30 ml) solution of 3-ethylaniline (1.63 g, 13.5 mmol) was added to a round-bottom flask containing 2,2-diphenylacetyl chloride (3.10 g, 13.5 mmol) and ammonium thiocyanate (1.03 g, 13.5 mmol). The mixture was refluxed for 2.5h then filtered off and left to evaporate at room temperature. The colourless precipitate obtained was washed with water and cold ethanol. Colourless crystals suitable for X-ray analysis were obtained by recrystallization of the precipitate in DMSO.

### Refinement

N-bound H atom were located in difference maps and refined freely, [N–H = 0.82 (3) and 0.90 (3) Å]. The remaining H atoms were positioned geometrically [C–H = 0.95–1.00 Å] and refined using a riding model with *U*_iso_(H) = 1.2 or 1.5 *U*_eq_(C). A rotating group model was applied to the methyl group. In the final refinement one outlier was omitted (1 2 0).

### Crystal data

**Table:**

C_23_H_22_N_2_OS	*F*(000) = 792
*M**_r_* = 374.49	*D*_x_ = 1.272 Mg m^−^^3^
Orthorhombic, *P**n**a*2_1_	Mo *K*α radiation, λ = 0.71073 Å
Hall symbol: P 2c -2n	Cell parameters from 2145 reflections
*a* = 10.0608 (2) Å	θ = 3.0–32.6°
*b* = 17.9092 (5) Å	µ = 0.18 mm^−^^1^
*c* = 10.8495 (3) Å	*T* = 100 K
*V* = 1954.87 (9) Å^3^	Plate, colourless
*Z* = 4	0.26 × 0.23 × 0.09 mm

### Data collection

**Table:**

Bruker SMART APEXII CCD area-detector diffractometer	4121 independent reflections
Radiation source: fine-focus sealed tube	2885 reflections with *I* > 2σ(*I*)
Graphite monochromator	*R*_int_ = 0.071
φ and ω scans	θ_max_ = 27.5°, θ_min_ = 2.2°
Absorption correction: multi-scan (*SADABS*; Bruker, 2009)	*h* = −13→12
*T*_min_ = 0.955, *T*_max_ = 0.983	*k* = −23→19
11560 measured reflections	*l* = −13→14

### Refinement

**Table:**

Refinement on *F*^2^	Secondary atom site location: difference Fourier map
Least-squares matrix: full	Hydrogen site location: inferred from neighbouring sites
*R*[*F*^2^ > 2σ(*F*^2^)] = 0.057	H atoms treated by a mixture of independent and constrained refinement
*wR*(*F*^2^) = 0.098	*w* = 1/[σ^2^(*F*_o_^2^) + (0.0368*P*)^2^] where *P* = (*F*_o_^2^ + 2*F*_c_^2^)/3
*S* = 0.99	(Δ/σ)_max_ < 0.001
4121 reflections	Δρ_max_ = 0.44 e Å^−^^3^
253 parameters	Δρ_min_ = −0.32 e Å^−^^3^
1 restraint	Absolute structure: Flack (1983), 1761 Friedel pairs
Primary atom site location: structure-invariant direct methods	Flack parameter: 0.17 (9)

### Special details

**Table:**

Experimental. The crystal was placed in the cold stream of an Oxford Cryosystems Cobra open-flow nitrogen cryostat (Cosier & Glazer, 1986) operating at 100.0 (1) K.
Geometry. All e.s.d.'s (except the e.s.d. in the dihedral angle between two l.s. planes) are estimated using the full covariance matrix. The cell e.s.d.'s are taken into account individually in the estimation of e.s.d.'s in distances, angles and torsion angles; correlations between e.s.d.'s in cell parameters are only used when they are defined by crystal symmetry. An approximate (isotropic) treatment of cell e.s.d.'s is used for estimating e.s.d.'s involving l.s. planes.
Refinement. Refinement of *F*^2^ against ALL reflections. The weighted *R*-factor *wR* and goodness of fit *S* are based on *F*^2^, conventional *R*-factors *R* are based on *F*, with *F* set to zero for negative *F*^2^. The threshold expression of *F*^2^ > σ(*F*^2^) is used only for calculating *R*-factors(gt) *etc*. and is not relevant to the choice of reflections for refinement. *R*-factors based on *F*^2^ are statistically about twice as large as those based on *F*, and *R*- factors based on ALL data will be even larger.

### Fractional atomic coordinates and isotropic or equivalent isotropic displacement parameters (Å^2^)

**Table:**

	*x*	*y*	*z*	*U*_iso_*/*U*_eq_	
S1	0.31660 (6)	0.86772 (5)	0.55685 (8)	0.0304 (2)	
O1	0.67282 (17)	0.76358 (11)	0.36156 (18)	0.0183 (5)	
N1	0.4543 (2)	0.79237 (14)	0.3925 (2)	0.0167 (6)	
N2	0.5813 (2)	0.84342 (13)	0.5464 (2)	0.0171 (6)	
C1	0.4471 (3)	0.60946 (17)	0.3775 (3)	0.0228 (8)	
H1A	0.5140	0.6275	0.4317	0.027*	
C2	0.3680 (3)	0.54945 (18)	0.4134 (3)	0.0273 (8)	
H2A	0.3806	0.5270	0.4919	0.033*	
C3	0.2716 (3)	0.52269 (19)	0.3353 (3)	0.0288 (9)	
H3A	0.2173	0.4819	0.3599	0.035*	
C4	0.2539 (3)	0.55506 (19)	0.2211 (3)	0.0267 (8)	
H4A	0.1879	0.5362	0.1667	0.032*	
C5	0.3322 (3)	0.61505 (17)	0.1855 (3)	0.0218 (7)	
H5A	0.3193	0.6373	0.1069	0.026*	
C6	0.4296 (3)	0.64289 (17)	0.2643 (3)	0.0159 (7)	
C7	0.5123 (3)	0.70924 (15)	0.2215 (3)	0.0151 (6)	
H7A	0.4535	0.7410	0.1689	0.018*	
C8	0.6323 (3)	0.68784 (16)	0.1434 (3)	0.0153 (7)	
C9	0.6652 (3)	0.73113 (17)	0.0418 (3)	0.0228 (7)	
H9A	0.6124	0.7734	0.0217	0.027*	
C10	0.7742 (3)	0.7136 (2)	−0.0309 (3)	0.0269 (8)	
H10A	0.7961	0.7440	−0.0997	0.032*	
C11	0.8512 (3)	0.65166 (19)	−0.0030 (3)	0.0260 (8)	
H11A	0.9252	0.6390	−0.0532	0.031*	
C12	0.8195 (3)	0.60861 (19)	0.0980 (3)	0.0289 (8)	
H12A	0.8725	0.5664	0.1180	0.035*	
C13	0.7102 (3)	0.62655 (18)	0.1710 (3)	0.0229 (7)	
H13A	0.6891	0.5964	0.2404	0.028*	
C14	0.5563 (3)	0.75715 (16)	0.3310 (3)	0.0133 (6)	
C15	0.4590 (3)	0.83515 (16)	0.5009 (3)	0.0161 (7)	
C16	0.6292 (3)	0.87797 (16)	0.6550 (3)	0.0174 (7)	
C17	0.7605 (3)	0.86083 (17)	0.6841 (3)	0.0228 (8)	
H17A	0.8072	0.8263	0.6336	0.027*	
C18	0.8250 (3)	0.89224 (18)	0.7834 (3)	0.0262 (8)	
C19	0.7545 (3)	0.94264 (19)	0.8556 (3)	0.0290 (8)	
H19A	0.7969	0.9658	0.9238	0.035*	
C20	0.6235 (3)	0.95954 (19)	0.8292 (3)	0.0279 (8)	
H20A	0.5763	0.9934	0.8805	0.034*	
C21	0.5598 (3)	0.92747 (17)	0.7284 (3)	0.0215 (7)	
H21A	0.4699	0.9395	0.7103	0.026*	
C22	0.9695 (3)	0.8747 (2)	0.8103 (4)	0.0425 (11)	
H22A	0.9778	0.8621	0.8989	0.051*	
H22B	1.0225	0.9204	0.7956	0.051*	
C23	1.0305 (3)	0.8111 (2)	0.7353 (3)	0.0440 (11)	
H23A	1.1230	0.8036	0.7608	0.066*	
H23B	1.0277	0.8239	0.6475	0.066*	
H23C	0.9799	0.7652	0.7495	0.066*	
H1N1	0.379 (3)	0.7853 (15)	0.366 (3)	0.019 (9)*	
H1N2	0.640 (3)	0.8181 (17)	0.500 (3)	0.025 (9)*	

### Atomic displacement parameters (Å^2^)

**Table:**

	*U*^11^	*U*^22^	*U*^33^	*U*^12^	*U*^13^	*U*^23^
S1	0.0143 (3)	0.0422 (5)	0.0348 (5)	0.0050 (4)	0.0033 (4)	−0.0157 (5)
O1	0.0112 (9)	0.0225 (12)	0.0212 (11)	0.0009 (9)	−0.0011 (10)	−0.0068 (10)
N1	0.0095 (12)	0.0191 (15)	0.0214 (15)	−0.0001 (11)	−0.0041 (12)	−0.0051 (12)
N2	0.0100 (11)	0.0222 (14)	0.0191 (13)	0.0018 (10)	0.0008 (13)	−0.0076 (14)
C1	0.0232 (17)	0.0190 (19)	0.0263 (19)	−0.0023 (14)	−0.0034 (15)	0.0008 (16)
C2	0.035 (2)	0.025 (2)	0.0213 (17)	0.0008 (16)	0.0088 (16)	0.0028 (17)
C3	0.0222 (16)	0.026 (2)	0.039 (2)	−0.0070 (14)	0.0067 (18)	0.0000 (18)
C4	0.0216 (16)	0.027 (2)	0.0316 (19)	−0.0109 (15)	−0.0075 (16)	−0.0021 (18)
C5	0.0221 (16)	0.025 (2)	0.0177 (16)	−0.0007 (14)	−0.0055 (14)	0.0040 (15)
C6	0.0173 (15)	0.0175 (18)	0.0130 (15)	0.0045 (13)	0.0037 (13)	−0.0005 (14)
C7	0.0157 (14)	0.0151 (16)	0.0144 (15)	0.0010 (12)	−0.0018 (13)	0.0006 (14)
C8	0.0161 (14)	0.0185 (17)	0.0114 (15)	−0.0049 (13)	−0.0016 (13)	−0.0035 (14)
C9	0.0164 (15)	0.0286 (18)	0.0233 (17)	−0.0023 (13)	−0.0078 (15)	0.0042 (17)
C10	0.0249 (17)	0.037 (2)	0.0183 (18)	−0.0127 (15)	0.0014 (15)	0.0005 (17)
C11	0.0171 (15)	0.035 (2)	0.0259 (19)	−0.0096 (15)	0.0067 (15)	−0.0141 (18)
C12	0.0235 (17)	0.0252 (19)	0.038 (2)	0.0031 (15)	0.0038 (17)	−0.0054 (17)
C13	0.0240 (16)	0.0197 (18)	0.0251 (18)	0.0020 (13)	0.0051 (14)	0.0009 (16)
C14	0.0157 (13)	0.0101 (16)	0.0140 (14)	−0.0014 (12)	0.0021 (13)	0.0044 (13)
C15	0.0142 (14)	0.0149 (17)	0.0192 (16)	−0.0001 (12)	0.0033 (13)	0.0034 (15)
C16	0.0176 (14)	0.0153 (17)	0.0192 (17)	−0.0038 (13)	0.0002 (14)	−0.0003 (15)
C17	0.0189 (15)	0.0226 (19)	0.0269 (18)	−0.0020 (14)	0.0027 (15)	−0.0025 (16)
C18	0.0278 (16)	0.0238 (19)	0.0269 (18)	−0.0064 (15)	−0.0091 (16)	0.0055 (17)
C19	0.044 (2)	0.025 (2)	0.0185 (16)	−0.0138 (17)	−0.0059 (17)	0.0016 (17)
C20	0.0384 (18)	0.025 (2)	0.0202 (17)	0.0013 (16)	0.0034 (17)	−0.0042 (16)
C21	0.0225 (15)	0.0230 (19)	0.0190 (16)	0.0021 (14)	0.0013 (15)	−0.0007 (16)
C22	0.036 (2)	0.038 (2)	0.053 (3)	−0.0038 (18)	−0.024 (2)	−0.004 (2)
C23	0.0202 (18)	0.086 (3)	0.025 (2)	0.004 (2)	−0.0051 (16)	0.003 (2)

### Geometric parameters (Å, º)

**Table:**

S1—C15	1.662 (3)	C9—H9A	0.9500
O1—C14	1.224 (3)	C10—C11	1.386 (5)
N1—C14	1.377 (3)	C10—H10A	0.9500
N1—C15	1.404 (4)	C11—C12	1.378 (4)
N1—H1N1	0.82 (3)	C11—H11A	0.9500
N2—C15	1.334 (3)	C12—C13	1.393 (4)
N2—C16	1.416 (4)	C12—H12A	0.9500
N2—H1N2	0.90 (3)	C13—H13A	0.9500
C1—C6	1.378 (4)	C16—C21	1.381 (4)
C1—C2	1.393 (4)	C16—C17	1.391 (4)
C1—H1A	0.9500	C17—C18	1.379 (4)
C2—C3	1.374 (4)	C17—H17A	0.9500
C2—H2A	0.9500	C18—C19	1.389 (5)
C3—C4	1.380 (5)	C18—C22	1.516 (4)
C3—H3A	0.9500	C19—C20	1.383 (4)
C4—C5	1.387 (4)	C19—H19A	0.9500
C4—H4A	0.9500	C20—C21	1.392 (4)
C5—C6	1.392 (4)	C20—H20A	0.9500
C5—H5A	0.9500	C21—H21A	0.9500
C6—C7	1.523 (4)	C22—C23	1.528 (5)
C7—C8	1.524 (4)	C22—H22A	0.9900
C7—C14	1.531 (4)	C22—H22B	0.9900
C7—H7A	1.0000	C23—H23A	0.9800
C8—C13	1.382 (4)	C23—H23B	0.9800
C8—C9	1.388 (4)	C23—H23C	0.9800
C9—C10	1.387 (4)		
			
C14—N1—C15	129.1 (2)	C11—C12—C13	120.4 (3)
C14—N1—H1N1	117 (2)	C11—C12—H12A	119.8
C15—N1—H1N1	114 (2)	C13—C12—H12A	119.8
C15—N2—C16	132.1 (3)	C8—C13—C12	120.5 (3)
C15—N2—H1N2	109.9 (18)	C8—C13—H13A	119.7
C16—N2—H1N2	117.7 (18)	C12—C13—H13A	119.7
C6—C1—C2	120.7 (3)	O1—C14—N1	122.6 (3)
C6—C1—H1A	119.6	O1—C14—C7	122.7 (2)
C2—C1—H1A	119.6	N1—C14—C7	114.7 (2)
C3—C2—C1	120.0 (3)	N2—C15—N1	113.7 (2)
C3—C2—H2A	120.0	N2—C15—S1	128.4 (2)
C1—C2—H2A	120.0	N1—C15—S1	117.9 (2)
C2—C3—C4	119.9 (3)	C21—C16—C17	119.4 (3)
C2—C3—H3A	120.0	C21—C16—N2	126.0 (3)
C4—C3—H3A	120.0	C17—C16—N2	114.5 (3)
C3—C4—C5	120.1 (3)	C18—C17—C16	122.3 (3)
C3—C4—H4A	119.9	C18—C17—H17A	118.9
C5—C4—H4A	119.9	C16—C17—H17A	118.9
C4—C5—C6	120.4 (3)	C17—C18—C19	117.7 (3)
C4—C5—H5A	119.8	C17—C18—C22	121.2 (3)
C6—C5—H5A	119.8	C19—C18—C22	121.0 (3)
C1—C6—C5	118.8 (3)	C20—C19—C18	120.8 (3)
C1—C6—C7	122.7 (3)	C20—C19—H19A	119.6
C5—C6—C7	118.5 (3)	C18—C19—H19A	119.6
C6—C7—C8	114.0 (2)	C19—C20—C21	120.7 (3)
C6—C7—C14	111.0 (2)	C19—C20—H20A	119.6
C8—C7—C14	110.1 (2)	C21—C20—H20A	119.6
C6—C7—H7A	107.1	C16—C21—C20	119.0 (3)
C8—C7—H7A	107.1	C16—C21—H21A	120.5
C14—C7—H7A	107.1	C20—C21—H21A	120.5
C13—C8—C9	118.7 (3)	C18—C22—C23	115.9 (3)
C13—C8—C7	121.9 (3)	C18—C22—H22A	108.3
C9—C8—C7	119.4 (3)	C23—C22—H22A	108.3
C10—C9—C8	120.9 (3)	C18—C22—H22B	108.3
C10—C9—H9A	119.5	C23—C22—H22B	108.3
C8—C9—H9A	119.5	H22A—C22—H22B	107.4
C11—C10—C9	120.0 (3)	C22—C23—H23A	109.5
C11—C10—H10A	120.0	C22—C23—H23B	109.5
C9—C10—H10A	120.0	H23A—C23—H23B	109.5
C12—C11—C10	119.5 (3)	C22—C23—H23C	109.5
C12—C11—H11A	120.3	H23A—C23—H23C	109.5
C10—C11—H11A	120.3	H23B—C23—H23C	109.5
			
C6—C1—C2—C3	0.4 (5)	C15—N1—C14—O1	−5.2 (5)
C1—C2—C3—C4	0.3 (5)	C15—N1—C14—C7	174.6 (3)
C2—C3—C4—C5	−0.7 (5)	C6—C7—C14—O1	114.1 (3)
C3—C4—C5—C6	0.3 (5)	C8—C7—C14—O1	−13.1 (4)
C2—C1—C6—C5	−0.8 (4)	C6—C7—C14—N1	−65.8 (3)
C2—C1—C6—C7	179.2 (3)	C8—C7—C14—N1	167.0 (2)
C4—C5—C6—C1	0.4 (4)	C16—N2—C15—N1	−176.0 (3)
C4—C5—C6—C7	−179.5 (3)	C16—N2—C15—S1	3.2 (5)
C1—C6—C7—C8	94.9 (3)	C14—N1—C15—N2	2.0 (4)
C5—C6—C7—C8	−85.2 (3)	C14—N1—C15—S1	−177.3 (2)
C1—C6—C7—C14	−30.1 (4)	C15—N2—C16—C21	−16.1 (5)
C5—C6—C7—C14	149.8 (3)	C15—N2—C16—C17	166.4 (3)
C6—C7—C8—C13	−40.4 (4)	C21—C16—C17—C18	−0.7 (4)
C14—C7—C8—C13	85.1 (3)	N2—C16—C17—C18	177.0 (3)
C6—C7—C8—C9	139.9 (3)	C16—C17—C18—C19	−0.1 (5)
C14—C7—C8—C9	−94.6 (3)	C16—C17—C18—C22	−177.8 (3)
C13—C8—C9—C10	−0.1 (4)	C17—C18—C19—C20	1.0 (5)
C7—C8—C9—C10	179.6 (3)	C22—C18—C19—C20	178.7 (3)
C8—C9—C10—C11	0.6 (4)	C18—C19—C20—C21	−1.2 (5)
C9—C10—C11—C12	−0.9 (4)	C17—C16—C21—C20	0.5 (4)
C10—C11—C12—C13	0.7 (4)	N2—C16—C21—C20	−176.8 (3)
C9—C8—C13—C12	−0.1 (4)	C19—C20—C21—C16	0.4 (4)
C7—C8—C13—C12	−179.8 (3)	C17—C18—C22—C23	−10.1 (5)
C11—C12—C13—C8	−0.2 (4)	C19—C18—C22—C23	172.3 (3)

### Hydrogen-bond geometry (Å, º)

**Table:**

*D*—H···*A*	*D*—H	H···*A*	*D*···*A*	*D*—H···*A*
N2—H1*N*2···O1	0.90 (3)	1.82 (3)	2.630 (3)	148 (3)
C21—H21*A*···S1	0.95	2.61	3.255 (3)	126
N1—H1*N*1···O1^i^	0.82 (3)	2.25 (3)	3.023 (3)	157 (3)
C7—H7*A*···*Cg*^ii^	1.00	2.87	3.844 (3)	166

Symmetry codes: (i) *x*−1/2, −*y*+3/2, *z*; (ii) −*x*−1/2, *y*+3/2, *z*+1/2.
